# Effects of Onion (*Allium cepa* L.) Extract Administration on Intestinal α-Glucosidases Activities and Spikes in Postprandial Blood Glucose Levels in SD Rats Model

**DOI:** 10.3390/ijms12063757

**Published:** 2011-06-08

**Authors:** Sun-Ho Kim, Sung-Hoon Jo, Young-In Kwon, Jae-Kwan Hwang

**Affiliations:** 1 Department of Biomaterials Science and Technology, Yonsei University, Seoul 120-749, Korea; E-Mail: info@nutraceutical.co.kr; 2 Department of Food and Nutrition, Hannam University, Daejeon 305-811, Korea; E-Mail: dutnskfk@hanmail.net; 3 Department of Biotechnology, College of Life Science and Biotechnology, Yonsei University, Seoul 120-749, Korea

**Keywords:** α-glucosidase, hyperglycemia, sucrase, postprandial spikes, *Allium cepa*

## Abstract

Diets high in calories and sweetened foods with disaccharides frequently lead to exaggerated postprandial spikes in blood glucose. This state induces immediate oxidant stress and free radicals which trigger oxidative stress-linked diabetic complications. One of the therapeutic approaches for decreasing postprandial hyperglycemia is to retard absorption of glucose by the inhibition of carbohydrate hydrolyzing enzymes, α-amylase and α-glucosidases, in the digestive organs. Therefore, the inhibitory activity of Korean onion (*Allium cepa* L.) extract against rat intestinal α-glucosidases, such as sucrase, maltase, and porcine pancreatic α-amylase were investigated *in vitro* and *in vivo*. The content of quercetin in ethyl alcohol extract of onion skin (EOS) was 6.04 g/100 g dried weight of onion skin. The *in vitro* half-maximal inhibitory concentrations (IC_50_) of EOS and quercetin, a major phenolic in onion, on rat intestinal sucrase were 0.40 and 0.11 mg/mL, respectively. The postprandial blood glucose lowering effects of EOS and quercetin were compared to a known type 2 diabetes drug (Acarbose), a strong α-glucosidase inhibitor in the Sprague-Dawley (SD) rat model. In rats fed on sucrose, EOS significantly reduced the blood glucose spike after sucrose loading. The area under the blood glucose-time curve (AUC_last_) in EOS-treated SD rats (0.5 g-EOS/kg) was significantly lower than in untreated SD rats (259.6 ± 5.1 *vs.* 283.1 ± 19.2 h·mg/dL). The AUC_last_ in quercetin-treated SD rats (0.5 g-quercetin/kg) was similar to in EOS-treated group (256.1 ± 3.2 *vs.* 259.6 ± 5.1 h·mg/dL). Results from this study indicates that although quercetin does have blood glucose lowering potential via α-glucosidase inhibition, there are other bioactive compounds present in onion skin. Furthermore, the effects of two weeks administration of EOS in a high carbohydrate-dietary mixture (Pico 5053) on sucrase and maltase activities in intestine were evaluated in SD rat model. Compared to the upper and middle parts of intestine, the activities of sucrase in the lower parts of intestine remained significantly higher after two weeks of EOS treatment. These results indicate that EOS may improve exaggerated postprandial spikes in blood glucose and glucose homeostasis since it inhibits intestinal sucrase and thus delays carbohydrate absorption, although clinical trials are needed.

## 1. Introduction

Recent studies indicate that about one-third of American adults and two-thirds of coronary artery disease (CAD) patients have abnormal glucose homeostasis [1,2]. The highly processed, calorie-dense, nutrient-depleted diet favored in the current American culture frequently leads to exaggerated postprandial spikes in blood glucose. This state, called postprandial hyperglycemia, induces immediate oxidant stress, which increases in direct proportion to the increase in glucose after a meal [3].

Hyperglycemia, a typical symptom in non-insulin dependent diabetes mellitus (NIDDM, type 2 diabetes) patients, is a condition characterized by a rapid rise in blood glucose levels and is due to hydrolysis of starch by pancreatic α-amylase and absorption of glucose in the small intestine by α-glucosidases [4]. The intestinal absorption of dietary carbohydrates such as maltose, sucrose, and starch is carried out by a group of α-glucosidases. One of the therapeutic approaches for decreasing postprandial hyperglycemia is to retard digestion of glucose by the inhibition of these carbohydrate hydrolyzing enzymes, α-glucosidases, in the digestive tract [4]. Therefore, inhibition of these carbohydrate-hydrolyzing enzymes can significantly decrease the postprandial hyperglycemia after a mixed carbohydrate diet and can be a key strategy in the control of diabetes mellitus [5].

Postprandial hyperglycemia has been linked to the onset of diabetic complications in NIDDM patients and triggers the generation of free radicals and oxidation-related damage in the retina, renal glomerulus and peripheral nerves [6,7]. Studies have shown that the glucose-induced increased levels of mitochondrial reactive oxygen species (ROS) produced by the mitochondrial electron transport chain seems to be the causal link between elevated levels of glucose and the pathways responsible for hyperglycemia-induced vascular complications [6,8]. One ongoing study, the European Study to Prevent Non-Insulin Dependent Diabetes (STOP-NIDDM), seems to show that treatment of people with impaired glucose tolerance (IGT) (based on postprandial blood sugars) with the drug acarbose (Precose® or Prandase®) helped prevent the onset of type 2 diabetes and reduced the risks of cardiovascular problems [9].

Important plant foods in traditional diet such as herbs, onion and beans have high phenolic phytochemicals. Among these plant foods Allium species, onions and garlic have long been used for a large range of purposes including medicine, nutrition, flavoring, condiment, foodstuff, and the treatment of common ailments as folk medicine [10]. Recent research has reported that phenolic phytochemicals from onion have blood glucose lowering effect and high antioxidant activity in alloxan-induced diabetic rat [11–13]. Further it is rich in flavonoids such as quercetin and sulfur compounds, such as allyl propyl disulfide that have perceived benefits to human health [14]. Epidemiological studies have also shown that the intake of certain types of flavonoids, including quercetin and myricetin is inversely associated with the risk of incident type 2 diabetes [15]. Quercetin, Isoquercitrin and rutin have shown inhibitory activities on α-glucosidase from the rat intestine [16]. Therefore, minimally processed and phenolic phytochemical enriched plants such as vegetables, fruits, nuts, seeds, and grains generally increase postprandial glucose and triglycerides to a lesser degree than do processed foods [17].

The effect of quercetin on hyperglycemia is already well-determined [16]. However, anti-hyperglycemic effect of onion skin extracts (EOS) and the long-term effects of EOS treatment on intestinal enzyme activities have not been investigated. The effects of EOS administration on the absorption of glucose via interruption and induction of intestinal α-glucosidases are also not known. Therefore, the aim of this study is to investigate the effects of EOS on hyperglycemia and long term EOS treatment in changes of the activities of small intestinal α-glucosidases on the brush border membranes. Clear knowledge of the mode of action of onion extract will contribute towards better understanding of the real effect of various onion products towards type 2 diabetes management. To determine the above, in this study, we (i) Prepared EOS by ethyl alcohol extraction; (ii) Compared the inhibitory activity of EOS to that of quercetin against α-amylase and α-glucosidases (anti-hyperglycemia potential); (iii) Evaluated the postprandial blood glucose lowering effect of EOS after sucrose loading in a Sprague-Dawley (SD) rat model; and (iv) Measured changes in intestinal enzyme activities after 2 weeks of EOS administration in SD rat model.

## 2. Results and Discussion

### 2.1. HPLC Analysis of EOS

Our previous studies have shown that the content of quercetin in onion skin extract has outstanding levels of quercetin comparing to the onion pulp [18]. Kim and Kim reported significant increase amounts of quercetin, 16.83, 2.67, 0.95, and 0.35 mg/g in the methyl alcohol extracts of onion skin, outer, middle and core parts respectively, with greater distance from the core [10].

Therefore, ethyl alcohol extract of onion skin (EOS) was prepared for further studies, and the major phenolic, quercetin was identified in the extracts using HPLC (Table 1). EOS had high concentrations of quercetin (6041.8 mg/100 g dried weight of onion skin). These results suggest that 95% ethyl alcohol (edible) extract of onion skin which has high quercetin content has the potential to contribute as a useful food application method for producing and enhancing bioactive food components such as quercetin in safety.

### 2.2. α-Amylase and α-Glucosidases Inhibition

The α-glucosidase inhibitors, which interfere with enzymatic action in the brush-border of the small intestine, could inhibit the liberation of d-glucose from oligosaccharides and disaccharides, resulting in delaying glucose absorption and decreasing postprandial plasma glucose levels [19]. Previous research with onion extracts reported that methyl alcohol extracts of onion had high microbial α-glucosidase (from Baker’s yeast) inhibitory activity [20]. It has been reported that most yeast α-glucosidase inhibitors did not show inhibitory activity against mammalian α-glucosidase due to the difference of molecular recognition in the binding site of the enzymes [21], Therefore, in order to have better health relevance, mammalian α-glucosidases (from rat intestine) were used to estimate the inhibitory activities of EOS and quercetin in this study. As a result, EOS had the highest sucrase inhibitory activity (0.40) followed by α-glucosidase (1.27), maltase (2.02), and α-amylase (IC_50_ of >3.00 mg/mL) (Table 2). The quercetin also showed potent sucrase inhibitory activity with an IC_50_ value of 0.11 mg/mL (Table 2), indicating a potential role as an active compound in onion extract. However, the content of quercetin in EOS was 6% of dried weight of onion skin (Table 1). This result suggests that sucrase inhibitory activity of EOS did not correlate with quercetin content and may be potentially linked to other unknown bioactive compounds in onion skin extract.

Based on these results, data trends for α-glucosidases inhibitory activities in EOS have important implications for the development and new design of onion-based functional foods.

### 2.3. *In Vivo* Blood Glucose Lowering Effect of EOS and Quercetin

EOS and quercetin showed significant inhibition against α-glucosidases especially for sucrase, which are membrane-bound enzymes at the epithelia of the small intestine and key enzymes of sucrose digestion (Table 2). Inhibition of sucrase may lead to a delayed and reduced rise in postprandial blood glucose levels.

**Figure 1:**
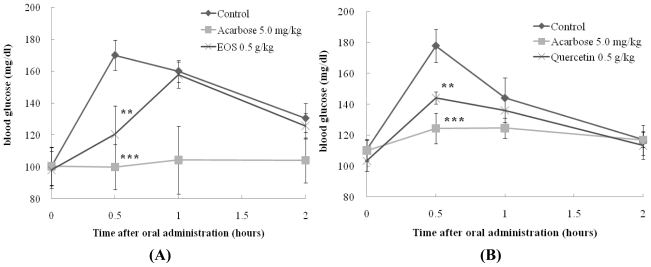
Effect of EOS **(A)** and quercetin **(B)** on sucrose loading test. After fasting for 24 h, 5-week-old, male SD rats were orally administered with sucrose solution (2.0 g/kg) with or without samples (EOS, Ethyl alcohol extract of onion skin: Positive control: Acarbose). Each point represents mean ± S.D. (n = 5). *p < 0.05, **p < 0.01, and ***p < 0.001 compared to different samples at the same concentration by unpaired Student’s *t*-test. **(A) (B)**

To confirm *in vitro* sucrase inhibitory activity of samples, the *in vivo* blood glucose reducing effects of EOS and its bioactive compound quercetin were evaluated with SD rats and the results are illustrated in Figure 1. In SD rats, EOS exerted a statistically significant decrease (*p* < 0.01) of the blood glucose at half an hour after sucrose loading. Quercetin significantly reduced (*p* < 0.01) the postprandial hyperglycemia caused by sucrose loading to an extent less than that observed in the acarbose administered group (*p* < 0.001) (Figure 1).

These data clearly demonstrate that the presence of phenolic phytochemicals such as quercetin in onion may play an important role in anti-hyperglycemic activity, and result in a reduction of blood glucose levels, which is in agreement with previous study by Kim *et al.* [10]. Furthermore, recent research has reported that phenolic phytochemicals from onion have high antioxidant activity in alloxan-induced diabetic rat [11–13]. Any dietary management of hyperglycemia linked to type 2 diabetes and related complications from oxidative dysfunction can benefit from specific enzyme inhibitory activity combined with antioxidant activity in the same whole food extracts. Insights from this study indicate that EOS have blood glucose lowering effect and high content of quercetin antioxidant and therefore have the potential to contribute to the reduction of hyperglycemia and oxidative stress-induced diabetic complications.

The pharmacokinetic parameters of SD rats administered with EOS, quercetin or acarbose are shown in Table 3. The EOS treatment at 0.5 g/kg body weight significantly decreased area under the blood glucose-time curve (AUC) (*p* < 0.001) and C_max_ (*p* < 0.01) blood glucose in rats that ingested sucrose compared to control. AUC glucose was lowest after acarbose. There was a decrease of 11% and 11% in EOS administration group compared to control group in the C_max_ and AUC_last_ respectively. The quercetin treatment at 0.5 g/kg body weight also significantly decreased blood glucose AUC (9.6%, *p* < 0.01) and C_max_ (19.1%, *p* < 0.05) blood glucose in rats that ingested sucrose. These data suggest that although quercetin has blood glucose reduction effect, there are possibly other bioactive compounds present in onion skins that contribute towards the observed blood glucose lowering effect of EOS.

On the other hand, the time of peak plasma concentration (T_max_) of glucose significantly increased in rats treated with EOS (1.0 h, *p* < 0.05) compared to control (0.6 h) when sucrose was orally administered to them (Table 3). These results may demonstrate the positive effects of EOS against postprandial spikes in blood glucose resulting from high sucrose ingestion and absorption. It suggests that EOS with high blood glucose lowering effect may be due to delaying absorption of glucose through inhibition of sucrase in the intestinal tract.

### 2.4. Sucrase and Maltase Activities in the Intestine

As shown in Figure 1(A), the blood glucose levels of the EOS-treated group were significantly lower than those of the control groups at 30 min of measurement, but not significant after 30 min. The time of peak plasma concentration (T_max_) of glucose significantly increased in rats treated with EOS (1.0 h, *p* < 0.05) compared to control (0.6 h) when sucrose was orally administered to them (Table 3). Therefore, it was hypothesized that EOS with high blood glucose lowering effect may be due to delaying absorption of glucose through inhibition of sucrase in the intestinal tract. The effects of two weeks administration of EOS in a high carbohydrate-dietary mixture (Pico 5053, Table 4) on sucrase and maltase activities in intestine were evaluated in SD rat model.

**Figure 2:**
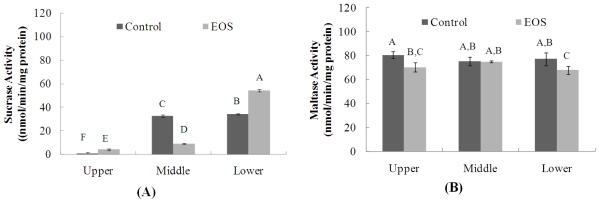
Effects of EOS administration on sucrase **(A)** and maltase **(B)** activities (nmol/min/mg protein) in different parts of intestine. The results represent the mean ± S.D. of values obtained from three measurements. Bar with different letters indicate statistical significance of differences among groups at *p* < 0.05 by Duncan’s test.

To prove intestinal α-glucosidases inhibition by EOS treatment, the sucrase and maltase activities in the intestinal tract were evaluated with SD rats and the results are illustrated in Figure 2. The activity of maltase was not inhibited by EOS administration (Figure 2B), but sucrase activity decreased in EOS-treated SD rats, and such decrease was found in the upper and middle of the three areas of the intestine (Figure 2A). Compared to the upper and middle parts of intestine, the activities of sucrase in the lower parts of intestine remained significantly higher after two weeks of EOS treatment, but no significant differences in maltase activities in whole part of intestine (Figure 2). These activities might be up-regulated by EOS, which are in agreement with previous study with voglibose α-glucosidase inhibitor by Yasuda *et al.* [22]. This suggests that two weeks treatment with EOS may increase the expression of sucrase in the lower part of intestine in compensation for the inhibition their activities in the upper and middle parts. Indeed modern studies indicate that Cinnamon a calorie-free herb rich, when added to a high-glycemic-index meal, significantly reduces the post-prandial glucose excursion, partly by slowing gastric emptying [23]. Recent study by Yasuda *et al.* also reported that the expressions of protein and mRNA of the sucrase-isomaltase (SI) complex were significantly higher in voglibose treated rats than in non-treated rats, especially in the lower part of the intestine [23].

Therefore, these results indicate that EOS may improve exaggerated postprandial spikes in blood glucose and glucose homeostasis due to the inhibition of sucrase in the upper and middle part of intestine and thus delays carbohydrate absorption, but such detailed relationship between EOS and the expression of SI complex should be examine in the future study.

## 3. Experimental Section

### 3.1. Materials

Korean onion (*Allium cepa* L.) was purchased from a local market in Daejeon, Korea. The dried skin of onion was identified by one of authors (Dr. Young-In Kwon). A voucher specimen (BFC O10012) was deposited at the Bioactive Food Components Lab. (BFCL) of the College of Life Science and Nano Technology, Hannam University. Porcine pancreatic α-amylase (EC 3.2.1.1), rat intestinal acetone powders of α-glucosidase (EC 3.2.1.20), quercetin (3,3′,4′,5,7-Pentahydroxyflavone dihydrate) and soluble starch (S9765-250G) were also purchased from Sigma-Aldrich Co. (St. Louis, MO, USA). Unless noted, all chemicals were purchased from Sigma-Aldrich Co. (St. Louis, MO, USA).

### 3.2. Preparation of Onion Extracts

After peeling of onion (*Allium cepa* L.) with a knife, 100 g of skin were mashed and stirred respectively in 1000 mL of 95% concentration of ethyl alcohol at 40 °C for 24 h. The skin extract (EOS) were then filtered through a Whatman # 2 filter, centrifuged at 7000 × g for 1 h, vacuum-evaporated at 45 °C, freeze-dried and kept at −70 °C until analysis.

### 3.3. HPLC Analysis of Quercetin

A volume of 2 mL of ethyl alchol extract of onion skin were filtered through a 0.2 μm syringe filter. The quercetin in the extract were analyzed using HPLC [24], (Tosoh 8010 series; Tosoh Corporation, Tokyo, Japan) equipped with a diode-array UV-vis detector (UV 8010; Tosoh Corporation) at 363 nm, and TSKgel-ODS 80 (15 cm × 7.6 mm) column (TSK ODS 80; Tosoh Corporation). The mobile phase used a water- acetonitrile mixture containing 0.05% phosphoric acid, where the flow rate and sample injection volume were fixed at 1.0 mL/min and 20 μL, respectively. The solvents used for gradient elution were (A) water:acetonitrile (95:5, v/v) and (B) water:acetonitrile (50:50, v/v). The solvent (B) was increased to 30% for the first 20 min and to 80% over the next 20 min, then decreased to 10% for the next 5 min and was maintained for the next 10 min (total run time, 55 min). As reference ingredient, pure standard of quercetin (purchased from Sigma Chemical Co.) in 100% methanol was used to calibrate the standard curve and retention times.

### 3.4. Preparation of Crude Enzyme Extracts

The small intestine was cut transversely into three segments (upper, middle, and lower part) of roughly equal length. Each segment was flushed with ice-cold phosphate buffered saline, frozen in liquid nitrogen, and stored at −80 °C. Each section of intestine was homogenized in 10 mL of 100 mM potassium phosphate buffer, pH 6.8, with a homogenizer (Ultra-Turrax T25, Janke & Kunkel Co., Staufen, Germany). After centrifugation at 3000 × g for 10 min, the supernatant obtained was used as crude enzyme solution.

### 3.5. α-Amylase Inhibition Assay

To evaluate the potency of onion (*Allium cepa* L.) extracts the dose dependency of onion skin extract (ES) and quercetin on α-amylase was measured using different concentrations (between 1.0 and 3.0 mg/mL). Porcine pancreatic α-amylase inhibition referred to the method of Kwon *et al.* [25]. Sample solution (200 μL) and 0.02 M sodium phosphate buffer (pH 6.9 with 0.006 M sodium chloride, 500 μL) containing α-amylase solution (0.5 mg/mL, 5.0 MU/mL) were incubated at 25 °C for 10 min. After pre-incubation, 500 μL of a 1% starch solution in 0.02 M sodium phosphate buffer was added. The reaction mixture was then incubated at 25 °C for 10 min. The reaction was stopped with 1.0 mL of dinitrosalicylic acid (DNS). The reaction mixture was then incubated in a boiling water bath for 5 min and cooled to room temperature. The reaction mixture was then diluted after adding distilled water, and absorbance was measured at 540 nm with ELISA micro-plate reader (SUNRISE; Tecan Trading AG, Saltzburg, Austria).

$$\text{Inhibition }(\%)=\left(\left[\frac{\Delta A_{540}^{Control}-\Delta A_{540}^{Extract}}{\left[\Delta A_{540}^{Control}\right]}\right]\right)\times 100$$

### 3.6. α-Glucosidase Inhibition Assay

To evaluate the potency of onion (*Allium cepa* L.) extracts, the dose dependency of onion skin extract (ES) and quercetin on rat intestinal α-glucosidase was measured using different concentrations (between 1.0 and 3.0 mg/mL). Rat intestinal α-glucosidase assay referred to the method of Kwon *et al*. [25] with slight modification. A total of 1 g of rat-intestinal acetone powder was suspended in 3 mL of 0.9% saline, and the suspension was sonicated 12 times for 30 s at 4 °C. After centrifugation (10,000 × g, 30 min, 4 °C), the resulting supernatant was used for the assay. Sample solution (50 μL) and 0.1 M phosphate buffer (pH 6.9, 100 μL) containing glucosidase solution (1.0 U/mL) was incubated at 25 °C for 10 min. After pre-incubation, 5 mM *p*-nitrophenyl-α-D-glucopyranoside solution (50 μL) in 0.1 M phosphate buffer (pH 6.9) was added to each well at timed intervals. The reaction mixtures were incubated at 25 °C for 5 min. Before and after incubation, absorbance was read at 405 nm and compared to a control which had 50 μL of buffer solution in place of the extract by micro-plate reader (SUNRISE; Tecan Trading AG, Saltzburg, Austria). The α-glucosidase inhibitory activity was expressed as inhibition % and was calculated as follows:

$$\text{Inhibition }(\%)=\left(\left[\frac{\Delta A_{405}^{Control}-\Delta A_{405}^{Extract}}{\left[\Delta A_{405}^{Control}\right]}\right]\right)\times 100$$

### 3.7. Maltase and Sucrase Activity Assay

Rat-intestinal crude enzyme (1.0 g) was suspended in 3 mL of 0.9% saline, and the suspension was sonicated twelve times for 30 s at 4 °C. After centrifugation (10,000 × g, 30 min, 4 °C), the resulting supernatant was used for the assay. Maltase and sucrase activities were assayed by modifying a method developed by Dahlqvist [26]. The activity was determined by incubating a solution of crude enzyme (50 μL), 0.1 M phosphate buffer (pH 7.0, 100 μL) containing 0.4 mg/mL sucrose or maltose at 37 °C for 30 min. The reaction mixture was heated in a boiling water bath to stop the reaction for 10 min, and then the amount of liberated glucose was measured by the glucose oxidase method [26].

### 3.8. Maltase and Sucrase Inhibition Assay

The crude enzyme solution prepared from rat intestinal acetone powder Sigma-Aldrich Co. (St. Louis, MO, USA) was used as the small intestinal maltase and sucrase, showing specific activities of 0.70 and 0.34 units/mL, respectively. Rat-intestinal acetone powder (1.0 g) was suspended in 3 mL of 0.9% saline, and the suspension was sonicated twelve times for 30 s at 4 °C. After centrifugation (10,000 × g, 30 min, 4 °C), the resulting supernatant was used for the assay. Maltase and sucrase inhibitory activities were assayed by modifying a method developed by Dahlqvist [26]. The inhibitory activity was determined by incubating a solution of an enzyme (50 μL), 0.1 M phosphate buffer (pH 7.0, 100 μL) containing 0.4 mg/mL sucrose or maltose, and a solution (50 μL) with various concentrations of sample solution (between 0.05 mM and 1.0 mM) at 37 °C for 30 min. The reaction mixture was heated in a boiling water bath to stop the reaction for 10 min, and then the amount of liberated glucose was measured by the glucose oxidase method [26]. The inhibitory activity was calculated from the formula as follows. Inhibition (%) = (C − T)/C × 100, where C is the enzyme activity without inhibitor and T is the enzyme activity with inhibitor.

### 3.9. Sugar Loading Test

Effect on hyperglycemia induced by carbohydrate loads in Sprague-Dawley (SD) rats was determined by the inhibitory action of EOS, quercetin and Acarbose on postprandial hyperglycemia. Five week-old male SD rats were purchased from Joongang Experimental Animal Co. (Seoul, Korea) and fed a solid diet (Samyang Diet Co., Seoul, Korea) for one week. The rats were housed in a ventilated room at 25 ± 2 °C with 50 ± 7% relative humidity, and under an alternating 12 h light/dark cycle. After 6 groups of 5 male SD rats (180–200 g) were fasted for 24 h, 2.0 g/kg of sucrose were orally administrated concurrently with 0–500 mg/kg inhibitors (EOS or quercetin or Acarbose). The blood samples were then taken from the tail after administration and blood glucose levels were measured at 0, 0.5, 1, and 2 h. The glucose level in blood was determined by glucose oxidase method and compared with that of the control group, which had not taken the inhibitors. The parameters for blood glucose levels were calculated using WinNonLin program (Version 5.2.1, Pharsight Corporation, Cary, NC, USA). Maximum observed peak blood glucose level (C_max_) and the time at which it is observed (T_max_) were determined based on the observed data. Area under the blood glucose-time curve up to the last sampled time-point (AUC_last_) was estimated by the trapezoidal rule.

### 3.10. Animal and Study Design

In this study, five SD rats were used under each condition. The animals were housed in individual cages in a room with a 12 h light/dark cycle (lights on from 06:00 h) with 50 ± 7% relative humidity. All rats were adapted to a meal-feeding schedule of free access to Pico 5053 diet (Table 4) (Oriental Bio. Co., Seongnam, Korea) with or without samples for 2 weeks. The experimental protocols were approved by the Institutional Animal Care and Use Committee (IACUC) of the Hannam University (Approval number: HNU2011-01). The rats had free access to tap water throughout the experiment. The rats were anesthetized with pentobarbital and killed, and blood was collected. The small intestine was cut transversely into three segments (upper, middle, and lower part) of roughly equal length. Each segment was flushed with ice-cold phosphate buffered saline, frozen in liquid nitrogen, and stored at −70 °C for measurement of enzyme activities.

### 3.11. Statistical Analysis

All data are presented as mean ± SD. Statistical analyses were carried out using the statistical package SPSS (Statistical Package for Social Science, SPSS Inc., Chicago, IL, USA) program and significance of each group was verified with the analysis of One-way ANOVA followed by the Duncan’s test of *p* < 0.05.

## 4. Conclusions

The modern calorie-dense, nutrient-poor diet of processed foods, especially when combined with a sedentary lifestyle and abdominal obesity, produces exaggerated postprandial increase in glucose and lipids, which leads to inflammation and atherosclerosis [3]. High-calorie meals rich in processed, easily digestible, quickly absorbable foods and drinks can lead to exaggerated postprandial elevations in blood glucose and triglycerides [3].

In contrast, a diet high in minimally processed, high-fiber, plant-based foods such as low glycemic index vegetables and fruits, whole grains, legumes, and nuts will markedly blunt the post-meal increase in glucose. Indeed modern studies indicate that vinegar significantly reduces post-meal glycemia, probably because acetic acid slows gastric emptying and thus delays carbohydrate absorption and improves satiety [27]. Improvements in diet exert profound and immediate favorable changes in the postprandial dysmetabolism.

EOS has a blood glucose lowering effect and high content of quercetin antioxidant and therefore has the potential to contribute to the reduction of hyperglycemia and oxidative stress-induced diabetic complications. These results show the positive effects of EOS against postprandial spikes in blood glucose resulting from high sucrose ingestion and absorption and demonstrate that the observed effect is not solely quercetin-dependent. Furthermore, our findings suggest that EOS with high blood glucose lowering effect may be due to delaying absorption of glucose through inhibition of sucrase in the intestinal tract.

These *in vitro* and *in vivo* studies therefore could provide the biochemical rationale for the benefit of onion-based dietary supplement and the basis for further clinical study.

Until today, quercetin was considered the most bioactive compound of onion skin. This research suggested that although quercetin does have blood glucose lowering potential via α-glucosidase inhibition, there are other bioactive compounds present in onion skin that contribute towards the observed bioactivities for type 2 diabetes management. Although, in this study, we provided evidence for EOS as a α-glucosidase inhibitor and its properties to decrease blood glucose, such detailed relationship between EOS and the expression of SI complex is not yet clear. Therefore, to provide the precise mechanism based on a single compound, further pharmacological and genetic studies are needed.

## Figures and Tables

**Table 1:** Quercetin content (mg/100 g-dried weight of onion skin) analyzed by HPLC in ethyl alcohol extracts of onion skin (EOS).

	Quercetin (mg/100 g-dried weight of onion skin)
EOS	6041.8

**Table 2:** Comparison of inhibitory activities (IC_50_) of EOS (mg/mL) and Quercetin (mg/mL) against α-glucosidase, α-amylase, sucrase, and maltase *in vitro*.

		IC_50_(mg/mL)
**EOS**	α-Glucosidase	1.27
	α-Amylase	>3.00
	Sucrase	0.40
	Maltase	2.02

**Quercetin**	α-Glucosidase	0.15
	α-Amylase	>0.60
	Sucrase	0.11
	Maltase	0.07

**Table 3:** Pharmacokinetic parameters of SD control rats or after administration of EOS, quercetin, and acarbose after sucrose ingestion.

		PK parameters		
		AUC_last_ (h·mg/dL)	C_max_ (mg/dL)	T_max_ (h)
**Sucrose**	Control	283.1 ± 19.2	177.8 ± 15.9	0.5 ± 0.0
	Acarbose (5.0 mg/kg)	230.7 ± 15.2**	128.4 ± 18.4**	0.7 ± 0.3
	Quercetin (0.5 g/kg)	256.1 ± 3.2*	144.0 ± 3.4**	0.5 ± 0.0

**Sucrose**	Control	290.6 ± 7.3	171.2 ± 7.2	0.6 ± 0.2
	Acarbose (5.0 mg/kg)	205.1 ± 29.4***	111.0 ± 15.8***	1.2 ± 0.8
	EOS(0.5 g/kg)	259.6± 5.1***	153.2 ± 9.3**	1.0 ± 0.0*

**Table 4:** Composition of the diets (g/kg).

	Control	EOS
Corn Starch	661	661
Casein	226	226
Soybean Oil	60	60
Vitamin Mix^1^	31	31
Mineral Mix^2^	9	9
Calcium Phospahte	10	10
Sodium chloride	3	3
Sample	0	100

1 AIN-93VX vitamin mix (Oriental Yeast Co., Tokyo, Japan).

2 AIN-93G mineral mix (Oriental Yeast Co., Tokyo, Japan).
